# Supplementary material for: The association between normal lung function and peak oxygen uptake in patients with exercise intolerance and coronary artery disease
Source: PLoS One. 2020 May 4;15(5):e0232693. doi: 10.1371/journal.pone.0232693 (PMC7197783; doi:10.1371/journal.pone.0232693)
Supplement: S4 Output — (HTM) [file pone.0232693.s008.htm]

xml version="1.0" encoding="UTF-8"?


S8\_output


|  |  |  |
| --- | --- | --- |
| IBM SPSS Web Report - S8\_output.spv     ---   Contents  Previous  Next  Help |  | Controls disabled by the system     --- |

- Regression

  - Descriptive Statistics
  - Correlations
  - Variables Entered/Removed
  - Model Summary
  - ANOVA
  - Coefficients
  - Residuals Statistics
  - Charts

    - \*zresid Histogram
    - \*zresid Normal P-P Plot
    - \*zresid by \*zpred Scatterplot

- Delete

Regression  
Regression - Descriptive Statistics - March 30, 2020

Descriptive StatisticsDescriptive Statistics, table, 1 levels of column headers and 1 levels of row headers, table with 4 columns and 11 rows

|  |  |  |  |
| --- | --- | --- | --- |
|  | Mean | Std. Deviation | N |
| VO2peak | 23.3276 | 6.15759 | 80 |
| TLCOoverVA\_zscore | -.03670 | .992445 | 80 |
| Sex | 1.24 | .428 | 80 |
| Age | 64.84 | 9.653 | 80 |
| BMI | 28.039875 | 4.0502060 | 80 |
| NeverVsFormer | .5750 | .49746 | 80 |
| NeverVsCurrent | .1875 | .39277 | 80 |
| Beta\_blocker | .65 | .480 | 80 |
| Inhaled\_bronchodilator | .18 | .382 | 80 |
|  |  |  |  |

Regression  
Regression - Correlations - March 30, 2020

CorrelationsCorrelations, table, 1 levels of column headers and 2 levels of row headers, table with 11 columns and 29 rows

|  |  |  |  |  |  |  |  |  |  |  |
| --- | --- | --- | --- | --- | --- | --- | --- | --- | --- | --- |
|  | | VO2peak | TLCOoverVA\_zscore | Sex | Age | BMI | NeverVsFormer | NeverVsCurrent | Beta\_blocker | Inhaled\_bronchodilator |
| Pearson Correlation | VO2peak | 1.000 | .168 | -.318 | -.415 | -.253 | -.051 | -.007 | -.116 | -.074 |
|  | TLCOoverVA\_zscore | .168 | 1.000 | -.039 | -.108 | .322 | -.134 | -.102 | .042 | -.079 |
|  | Sex | -.318 | -.039 | 1.000 | .062 | -.059 | -.114 | -.042 | .040 | .284 |
|  | Age | -.415 | -.108 | .062 | 1.000 | -.181 | .099 | -.216 | -.042 | -.078 |
|  | BMI | -.253 | .322 | -.059 | -.181 | 1.000 | -.021 | -.009 | .017 | -.034 |
|  | NeverVsFormer | -.051 | -.134 | -.114 | .099 | -.021 | 1.000 | -.559 | -.154 | .063 |
|  | NeverVsCurrent | -.007 | -.102 | -.042 | -.216 | -.009 | -.559 | 1.000 | .218 | .032 |
|  | Beta\_blocker | -.116 | .042 | .040 | -.042 | .017 | -.154 | .218 | 1.000 | .131 |
|  | Inhaled\_bronchodilator | -.074 | -.079 | .284 | -.078 | -.034 | .063 | .032 | .131 | 1.000 |
| Sig. (1-tailed) | VO2peak | . | .069 | .002 | .000 | .012 | .325 | .477 | .153 | .257 |
|  | TLCOoverVA\_zscore | .069 | . | .365 | .170 | .002 | .118 | .185 | .357 | .242 |
|  | Sex | .002 | .365 | . | .294 | .301 | .156 | .355 | .362 | .005 |
|  | Age | .000 | .170 | .294 | . | .054 | .192 | .027 | .354 | .246 |
|  | BMI | .012 | .002 | .301 | .054 | . | .428 | .469 | .442 | .381 |
|  | NeverVsFormer | .325 | .118 | .156 | .192 | .428 | . | .000 | .087 | .289 |
|  | NeverVsCurrent | .477 | .185 | .355 | .027 | .469 | .000 | . | .026 | .390 |
|  | Beta\_blocker | .153 | .357 | .362 | .354 | .442 | .087 | .026 | . | .123 |
|  | Inhaled\_bronchodilator | .257 | .242 | .005 | .246 | .381 | .289 | .390 | .123 | . |
| N | VO2peak | 80 | 80 | 80 | 80 | 80 | 80 | 80 | 80 | 80 |
|  | TLCOoverVA\_zscore | 80 | 80 | 80 | 80 | 80 | 80 | 80 | 80 | 80 |
|  | Sex | 80 | 80 | 80 | 80 | 80 | 80 | 80 | 80 | 80 |
|  | Age | 80 | 80 | 80 | 80 | 80 | 80 | 80 | 80 | 80 |
|  | BMI | 80 | 80 | 80 | 80 | 80 | 80 | 80 | 80 | 80 |
|  | NeverVsFormer | 80 | 80 | 80 | 80 | 80 | 80 | 80 | 80 | 80 |
|  | NeverVsCurrent | 80 | 80 | 80 | 80 | 80 | 80 | 80 | 80 | 80 |
|  | Beta\_blocker | 80 | 80 | 80 | 80 | 80 | 80 | 80 | 80 | 80 |
|  | Inhaled\_bronchodilator | 80 | 80 | 80 | 80 | 80 | 80 | 80 | 80 | 80 |
|  |  |  |  |  |  |  |  |  |  |  |

Regression  
Regression - Variables Entered/Removed - March 30, 2020

Variables Entered/RemovedaVariables Entered/Removed, table, 1 levels of column headers and 1 levels of row headers, table with 4 columns and 5 rows

|  |  |  |  |
| --- | --- | --- | --- |
| Model | Variables Entered | Variables Removed | Method |
| 1 | Inhaled\_bronchodilator, NeverVsCurrent, BMI, Beta\_blocker, Sex, Age, TLCOoverVA\_zscore, NeverVsFormerb | . | Enter |
|  |  |  |  |
| --- | --- | --- | --- |
| a. Dependent Variable: VO2peak | | | |
| b. All requested variables entered. | | | |
|  |  |  |  |

Regression  
Regression - Model Summary - March 30, 2020

Model SummarybModel Summary, table, 1 levels of column headers and 1 levels of row headers, table with 5 columns and 5 rows

|  |  |  |  |  |
| --- | --- | --- | --- | --- |
| Model | R | R Square | Adjusted R Square | Std. Error of the Estimate |
| 1 | .682a | .465 | .404 | 4.75190 |
|  |  |  |  |  |
| --- | --- | --- | --- | --- |
| a. Predictors: (Constant), Inhaled\_bronchodilator, NeverVsCurrent, BMI, Beta\_blocker, Sex, Age, TLCOoverVA\_zscore, NeverVsFormer | | | | |
| b. Dependent Variable: VO2peak | | | | |
|  |  |  |  |  |

Regression  
Regression - ANOVA - March 30, 2020

ANOVAaANOVA, table, 1 levels of column headers and 2 levels of row headers, table with 7 columns and 7 rows

|  |  |  |  |  |  |  |  |
| --- | --- | --- | --- | --- | --- | --- | --- |
| Model | | Sum of Squares | df | Mean Square | F | Sig. |  |
| 1 | Regression | 1392.141 | 8 | 174.018 | 7.707 | .000b |  |
|  | Residual | 1603.219 | 71 | 22.581 |  |  |  |
|  | Total | 2995.360 | 79 |  |  |  |  |
|  |  |  |  |  |  |  |  |
| --- | --- | --- | --- | --- | --- | --- | --- |
| a. Dependent Variable: VO2peak | | | | | | |  |
| b. Predictors: (Constant), Inhaled\_bronchodilator, NeverVsCurrent, BMI, Beta\_blocker, Sex, Age, TLCOoverVA\_zscore, NeverVsFormer | | | | | | |  |
|  |  |  |  |  |  |  |  |

Regression  
Regression - Coefficients - March 30, 2020

CoefficientsaCoefficients, table, 2 levels of column headers and 2 levels of row headers, table with 9 columns and 13 rows

|  |  |  |  |  |  |  |  |  |  |
| --- | --- | --- | --- | --- | --- | --- | --- | --- | --- |
| Model | | Unstandardized Coefficients | | Standardized Coefficients | t | Sig. | 95.0% Confidence Interval for B | |  |
|  |  | B | Std. Error | Beta |  |  | Lower Bound | Upper Bound |  |
| 1 | (Constant) | 69.282 | 6.298 |  | 11.000 | .000 | 56.723 | 81.840 |  |
|  | TLCOoverVA\_zscore | 1.338 | .594 | .216 | 2.254 | .027 | .154 | 2.522 |  |
|  | Sex | -4.677 | 1.339 | -.325 | -3.493 | .001 | -7.347 | -2.007 |  |
|  | Age | -.302 | .058 | -.474 | -5.181 | .000 | -.419 | -.186 |  |
|  | BMI | -.653 | .141 | -.429 | -4.618 | .000 | -.935 | -.371 |  |
|  | NeverVsFormer | -1.522 | 1.371 | -.123 | -1.110 | .271 | -4.255 | 1.212 |  |
|  | NeverVsCurrent | -2.334 | 1.770 | -.149 | -1.319 | .191 | -5.864 | 1.195 |  |
|  | Beta\_blocker | -1.445 | 1.155 | -.113 | -1.251 | .215 | -3.749 | .858 |  |
|  | Inhaled\_bronchodilator | .180 | 1.497 | .011 | .120 | .905 | -2.806 | 3.166 |  |
|  |  |  |  |  |  |  |  |  |  |
| --- | --- | --- | --- | --- | --- | --- | --- | --- | --- |
| a. Dependent Variable: VO2peak | | | | | | | | |  |
|  |  |  |  |  |  |  |  |  |  |

Regression  
Regression - Residuals Statistics - March 30, 2020

Residuals StatisticsaResiduals Statistics, table, 1 levels of column headers and 1 levels of row headers, table with 6 columns and 7 rows

|  |  |  |  |  |  |
| --- | --- | --- | --- | --- | --- |
|  | Minimum | Maximum | Mean | Std. Deviation | N |
| Predicted Value | 13.8428 | 33.9686 | 23.3276 | 4.19786 | 80 |
| Residual | -10.82363 | 14.35838 | .00000 | 4.50488 | 80 |
| Std. Predicted Value | -2.259 | 2.535 | .000 | 1.000 | 80 |
| Std. Residual | -2.278 | 3.022 | .000 | .948 | 80 |
|  |  |  |  |  |  |
| --- | --- | --- | --- | --- | --- |
| a. Dependent Variable: VO2peak | | | | | |
|  |  |  |  |  |  |

Charts  
Charts - \*zresid Histogram - March 30, 2020

Charts  
Charts - \*zresid Normal P-P Plot - March 30, 2020

Charts  
Charts - \*zresid by \*zpred Scatterplot - March 30, 2020

{"copyright":"(C) Copyright IBM Corp. 2011","grammar":[{"elements":[{"data":{"$ref":"dSource"},"style":{"symbol":"circle","outline":{"r":0,"b":157,"g":100},"size":6.6666665,"fill":{"r":119,"b":119,"g":118}},"position":[{"field":{"$ref":"fVariable1"}},{"field":{"$ref":"fVariable"}}],"type":"point"}],"coordinates":{"style":{"outline":{"r":0,"b":157,"g":100},"fill":{"r":255,"b":255,"g":255}},"dimensions":[{"scale":{"padding":{"left":"5%","right":"5%"}},"axis":[{"tickStyle":{"padding":5.0,"fill":{"r":0,"b":157,"g":100},"font":{"size":"8pt","weight":"normal","family":"sans-serif"}},"gridStyle":{"fill":{"r":0,"b":157,"g":100}},"lineStyle":{"fill":{"r":0,"b":157,"g":100},"stroke":{"width":0.6666667}},"titleStyle":{"padding":6.0,"fill":{"r":0,"b":157,"g":100},"font":{"size":"12pt","weight":"bold","family":"sans-serif"}},"title":["Regression Standardized Residual"],"markStyle":{"fill":{"a":0,"r":0,"b":157,"g":100},"stroke":{"width":1.3333334}}}]},{"scale":{"padding":{"left":"5%","right":"5%"}},"axis":[{"tickStyle":{"padding":5.0,"fill":{"r":0,"b":157,"g":100},"font":{"size":"8pt","weight":"normal","family":"sans-serif"}},"gridStyle":{"fill":{"r":0,"b":157,"g":100}},"lineStyle":{"fill":{"r":0,"b":157,"g":100},"stroke":{"width":0.6666667}},"titleStyle":{"padding":6.0,"fill":{"r":0,"b":157,"g":100},"font":{"size":"12pt","weight":"bold","family":"sans-serif"}},"title":["Regression Standardized Predicted Value"],"markStyle":{"fill":{"a":0,"r":0,"b":157,"g":100},"stroke":{"width":1.3333334}}}]}]}}],"data":[{"id":"dSource","fields":[{"min":-2.259429059091126,"max":2.534871768600855,"id":"fVariable","label":"X Variable\_1"},{"min":-2.277748286787788,"max":3.021608375218541,"id":"fVariable1","label":"Y Axis"},{"min":1.0,"max":92.0,"id":"fVariable2","label":"Case Number"}],"rows":[[0.4773450296796992,2.307218402085732,1],[-2.218866646508905,1.819565304664356,2],[-0.6187911077384548,-0.1868773897575775,3],[0.01077625053103714,0.9634021206248183,4],[-1.207042298638876,0.1598948320501141,5],[0.562830101139885,-0.8966456648527911,6],[0.4794136232312459,-1.683979710177994,7],[-1.860551318009053,0.4637306882990594,8],[0.4897889718530958,-0.752033104931916,9],[0.1130381586195507,-0.6989073295787273,10],[0.1970727386814869,2.844509192195673,11],[-1.394552941734961,-0.2258051298688631,13],[2.534871768600855,-0.2307877480962011,14],[-0.9001944100949777,-0.2002514045002574,15],[0.1696440957518454,-0.7200023350081298,16],[1.417273280997864,0.3536801313395356,17],[-0.7923709547797329,0.4432212051704272,18],[0.1702197920736162,1.044571750067755,21],[1.167166548612459,0.2949024414741029,22],[-1.533783999045248,-0.2067994135731952,23],[-1.094152995223979,0.0818999678643842,24],[-0.216659876892036,0.8386840856263834,25],[0.7658895519291667,1.259990921566704,26],[-2.005154160900652,0.4882958027137418,28],[1.116358093016062,0.5179917282194711,29],[0.4958378355038385,-0.2859093465509255,31],[-1.035469580570062,-0.7975612483952281,32],[0.8531936803252209,-0.219688529335488,33],[-0.3386391645217996,1.141808735481379,34],[-0.283435090574518,-0.5334110812862515,35],[-0.2782732125559868,-0.2430484683718592,36],[0.3022978700290068,1.804643443363769,38],[1.817620172817261,3.021608375218541,39],[-0.301559547874017,-0.9447657854359015,40],[-0.2923576855330282,0.6354890677795613,41],[-0.5988781690880958,-0.8721190133101189,42],[-0.3684647193371363,0.1459387638937006,43],[1.109721228485785,-0.7178382021587038,44],[0.7963840927488094,-0.08157485064115053,45],[-1.146485937151521,-0.3364728425484796,46],[1.086944605006,-0.09112311971778804,47],[1.081119578273425,0.2757248343231041,48],[-0.2113595168578405,0.7037419257848309,50],[0.2387701167911534,-0.6968102256856202,51],[1.164493557467743,-0.1293353459276642,53],[-0.05715667175524852,0.9518959827545195,54],[-0.09185147719216614,-0.8446412214536214,55],[1.39923042726517,0.3927130576181831,57],[-1.530263290360591,0.7452758686742785,59],[0.4805570370338464,-1.588940688272037,60],[0.8908546643236824,1.134018547013252,61],[0.887245920437631,-0.1924567146019808,62],[1.572310917295258,-0.04413402331482637,64],[0.5511905719680379,0.1011617404640493,65],[1.456342950282483,-2.277748286787788,66],[-0.7087466217725297,-0.557355544955986,67],[-0.1915641757229844,-0.1665440363687762,68],[-1.136211753800418,-0.2578133852380156,69],[0.6229979566929983,-0.5610630770648846,70],[0.2728389314002914,0.005183575647063658,71],[-0.001754932793635577,0.4828915215671727,72],[-0.4317858368423795,0.8056678864417043,73],[-0.4006133824913875,-1.905843233952918,74],[-0.2917118705535193,-0.06572719006983228,75],[0.111334444151122,0.8131330741762637,76],[0.8123273850000123,-1.248592547163548,77],[-1.097564237036724,0.6805175518412033,78],[-0.708707726221244,0.3623829983649591,79],[-0.2286639122062377,-0.8917540920230731,80],[-1.251704125489305,-1.01389362606687,81],[-1.059096306238102,-0.6385446255807756,82],[-1.169335944876282,-0.7040688965374867,83],[-0.5066960602709935,-0.7281588337354122,84],[0.2172578557885746,-0.09613910058000091,85],[0.2225038944184642,-0.475643878078386,86],[-2.259429059091126,-0.179655779822012,87],[1.588703798544754,-0.2179172149991729,88],[1.57628797835401,0.1457009483608014,90],[0.9041614964063759,-1.120180203449758,91],[-0.3643162531831094,-0.7024929829026684,92]]}],"size":{"width":850.0,"height":500.0},"style":{"outline":{"a":0.0,"r":0,"b":0,"g":0},"fill":{"r":255,"b":255,"g":255}},"titles":[{"backgroundStyle":{"outline":{"a":0.0,"r":0,"b":0,"g":0},"fill":{"a":0.0,"r":0,"b":0,"g":0}},"style":{"padding":3.0,"fill":{"r":0,"b":157,"g":100},"font":{"size":"12pt","weight":"bold","family":"sans-serif"}},"type":"title","content":["Scatterplot"]},{"backgroundStyle":{"outline":{"a":0.0,"r":0,"b":0,"g":0},"fill":{"a":0.0,"r":0,"b":0,"g":0}},"style":{"padding":3.0,"fill":{"r":0,"b":157,"g":100},"font":{"size":"12pt","weight":"bold","family":"sans-serif"}},"type":"title","content":["Dependent Variable: VO2peak"]}],"version":"6.0"}

IBM SPSS Web Report

X

ABOUT

|  |
| --- |
| Created Using: IBM SPSS Statistics 25 |
| Creation Date: Mar 30, 2020 |
| Document Version: OriginalSaved Copy |
| Saved Date:  Mar 30, 2020 |

Navigation Controls

|  |
| --- |
| Contents - Opens and closes the list of charts and tables in the Web Report |
| Next & Previous - Display the next or previous table or chart in the Web Report |
| Help - Opens Help |

Toolbar Buttons

|  |  |
| --- | --- |
|  | Undo - Undoes the last change in the document. |
|  | Edit - Open the Editor tool for tables and charts. Certain editing options are only available when you are connected to an Internet server. |
|  | Save - Creates a new copy of the Web Report with the saved changes. |
|  | Print - Prints the current object when in Object View and all objects in Page View. |
|  | Page View - Switches the Web Report to display all the tables and charts on a single page. |
|  | Object View - Switches the Web Report so that each table or chart is displayed one at a time. |

Connecting to a Server

:   The status of the Web Report's connection to an Internet server appears in the top right corner of the Web Report.
:   An Internet connection is not required to open a Web Report. With a saved copy of the Web Report you can view all of the charts and tables, and have some limited editing ability, when not connected to the Internet.
:   Connecting a Web Report to an Internet server will enable far greater editing capabilities for tables and for charts.

- If the author specified an Internet server when they created the Web Report, the Web Report will attempt to connect to the server automatically when it is opened.
- If the Web Report does not connect to a server, click on the server Status Message to open tools to retry the connection, try a different server, or enter a new server address.
- For information about adding the enhanced controls to your Internet Server, go to https://developer.ibm.com/predictiveanalytics.
- If you specify a new server connection, the preferred format is http://xxx.xxx.xxx.xxx:xxxx.

Editing Tables

|  |  |
| --- | --- |
| Some of this functionality is only available when connected to an Internet server. | |
|  | Create a chart - Create a chart from the selected cells in the table. |
|  | Pivot and Sort - Transpose, sort, and pivot the table. |
|  | Background color - The background color of the selected cells. |
|  | Text Color and Style - Font color, style, and size. |
|  | Number Format - Font color, style, and size. |

Editing Charts

|  |  |
| --- | --- |
| All of this functionality is only available when connected to an Internet server. | |
|  | Chart Size - Change the height and width of the chart |
|  | Background color - The background color of the selected object. |
|  | Border and Line Style - The color and thickness of the line or border. |
|  | Text Color and Style - Font color, style, and size. |
|  | Number Format - Font color, style, and size. |
|  | Axis Properties - Change the scale and display axis titles and ticks. |

Save

X
New Name  
   
  
What to Save   

Save the entire document  
Only save the current object

Server Connection

X
  
Saved Server Connections  
   
  
  
  
Status

Add a chart

Pivot and Sort

Chart Size   
  

|  |  |  |
| --- | --- | --- |
|  |  |  |
|  |  |  |
| Lock aspect ratio | | |

Background   

|  |  |  |  |  |  |
| --- | --- | --- | --- | --- | --- |
|  | |  | |  | |
|  |  |  |  |  |  |
|  |  |  |  |  |  |
|  |  |  |  |  |  |

Line and Borders   

|  |  |  |  |  |  |
| --- | --- | --- | --- | --- | --- |
|  | |  | |  | |
|  |  |  |  |  |  |
|  |  |  |  |  |  |
|  |  |  |  |  |  |

Text Format   

|  |  |  |  |  |  |
| --- | --- | --- | --- | --- | --- |
|  | |  | |  | |
|  |  |  |  |  |  |
|  |  |  |  |  |  |
|  |  |  |  |  |  |

  

|  |  |  |
| --- | --- | --- |
|  |  |  |

  

|  |  |  |  |
| --- | --- | --- | --- |
|  |  |  | Font Family  Abadi MT Condensed Extra Bold Abadi MT Condensed Light Al Bayan Al Nile Al Tarikh American Typewriter Andale Mono Apple Braille Apple Chancery Apple Color Emoji Apple SD Gothic Neo Apple Symbols AppleGothic AppleMyungjo Arial Arial Black Arial Hebrew Arial Hebrew Scholar Arial Narrow Arial Rounded MT Bold Arial Unicode MS Athelas Avenir Avenir Next Avenir Next Condensed Ayuthaya Baghdad Bangla MN Bangla Sangam MN Baskerville Baskerville Old Face Batang Bauhaus 93 Beirut Bell MT Bernard MT Condensed Big Caslon Bodoni 72 Bodoni 72 Oldstyle Bodoni 72 Smallcaps Bodoni Ornaments Book Antiqua Bookman Old Style Bookshelf Symbol 7 Bradley Hand Braggadocio Britannic Bold Brush Script MT Calibri Calisto MT Cambria Cambria Math Candara Century Century Gothic Century Schoolbook Chalkboard Chalkboard SE Chalkduster Charter Cochin Colonna MT Comic Sans MS Consolas Constantia Cooper Black Copperplate Copperplate Gothic Bold Copperplate Gothic Light Corbel Corsiva Hebrew Courier Courier New Curlz MT Damascus DecoType Naskh Desdemona Devanagari MT Devanagari Sangam MN Dialog DialogInput Didot DIN Alternate DIN Condensed Diwan Kufi Diwan Thuluth Edwardian Script ITC Engravers MT Euphemia UCAS Eurostile Farah Farisi Footlight MT Light Franklin Gothic Book Franklin Gothic Medium Futura Gabriola Garamond GB18030 Bitmap Geeza Pro Geneva Georgia Gill Sans Gill Sans MT Gloucester MT Extra Condensed Goudy Old Style Gujarati MT Gujarati Sangam MN Gulim Gurmukhi MN Gurmukhi MT Gurmukhi Sangam MN Haettenschweiler Harrington Heiti SC Heiti TC Helvetica Helvetica Neue HelvNeue Roman for IBM Herculanum Hiragino Kaku Gothic Pro Hiragino Kaku Gothic ProN Hiragino Kaku Gothic Std Hiragino Kaku Gothic StdN Hiragino Maru Gothic Pro Hiragino Maru Gothic ProN Hiragino Mincho Pro Hiragino Mincho ProN Hiragino Sans Hiragino Sans GB Hoefler Text Impact Imprint MT Shadow InaiMathi Iowan Old Style ITF Devanagari ITF Devanagari Marathi Kailasa Kannada MN Kannada Sangam MN Kefa Khmer MN Khmer Sangam MN Kino MT Kohinoor Bangla Kohinoor Devanagari Kohinoor Telugu Kokonor Krungthep KufiStandardGK Lao MN Lao Sangam MN Lucida Blackletter Lucida Bright Lucida Calligraphy Lucida Console Lucida Fax Lucida Grande Lucida Handwriting Lucida Sans Lucida Sans Typewriter Lucida Sans Unicode Luminari Malayalam MN Malayalam Sangam MN Marion Marker Felt Marlett Matura MT Script Capitals Meiryo Menlo Microsoft Himalaya Microsoft Sans Serif Microsoft Tai Le Microsoft Yi Baiti MingLiU MingLiU-ExtB MingLiU\_HKSCS MingLiU\_HKSCS-ExtB Mishafi Mishafi Gold Mistral Modern No. 20 Monaco Mongolian Baiti Monospaced Monotype Corsiva Monotype Sorts MS Gothic MS Mincho MS PGothic MS PMincho MS Reference Sans Serif MS Reference Specialty Mshtakan MT Extra Muna Myanmar MN Myanmar Sangam MN Nadeem New Peninim MT News Gothic MT Noteworthy Noto Nastaliq Urdu Onyx Optima Oriya MN Oriya Sangam MN Osaka Palatino Palatino Linotype Papyrus Perpetua Perpetua Titling MT Phosphate PingFang HK PingFang SC PingFang TC Plantagenet Cherokee Playbill PMingLiU PMingLiU-ExtB PT Mono PT Sans PT Sans Caption PT Sans Narrow PT Serif PT Serif Caption Raanana Rockwell Rockwell Extra Bold Sana SansSerif Sathu Savoye LET Seravek Serif Shree Devanagari 714 SignPainter Silom SimHei SimSun SimSun-ExtB Sinhala MN Sinhala Sangam MN Skia Snell Roundhand Songti SC Songti TC Stencil STHeiti STIXGeneral STIXIntegralsD STIXIntegralsSm STIXIntegralsUp STIXIntegralsUpD STIXIntegralsUpSm STIXNonUnicode STIXSizeFiveSym STIXSizeFourSym STIXSizeOneSym STIXSizeThreeSym STIXSizeTwoSym STIXVariants STSong Sukhumvit Set Superclarendon Symbol Tahoma Tamil MN Tamil Sangam MN Telugu MN Telugu Sangam MN Thonburi Times Times New Roman Trattatello Trebuchet MS Tw Cen MT Verdana Waseem Webdings Wide Latin Wingdings Wingdings 2 Wingdings 3 Zapf Dingbats Zapfino |

Number Format   
  

|  |  |  |
| --- | --- | --- |
| 0.00 |  |  |

Axis Options   
  

|  |  |  |
| --- | --- | --- |
|  |  |  |
|  |  |  |
| Display Axis Title | | | |
| Display Ticks | | | |
